# The effect of one‐lung ventilation on sentinel lymph node mapping in dog lungs

**DOI:** 10.1111/vsu.70145

**Published:** 2026-08-25

**Authors:** Sarah J. Marvel, Nontalie Kaschark, Pedro Boscan, Eric Monnet

**Affiliations:** ^1^ Department of Clinical Sciences, College of Veterinary Medicine and Biomedical Sciences Colorado State University Fort Collins Colorado USA

## Abstract

**Objective:**

To determine the sentinel lymph node (SLN) for the caudal lung lobe using indocyanine green (ICG) and near infra‐red fluorescence (NIRF) in normal dogs. The secondary objective was to determine the effect of one lung ventilation (OLV) on time to SLN detection.

**Study design:**

Prospective, randomized experimental study.

**Animals:**

Purpose‐bred Beagles (*n* = 21).

**Methods:**

Dogs were randomized to undergo OLV or normal ventilation (NV) and to undergo right or left caudal lung injections with ICG through lateral intercostal thoracoscopy. The draining lymph nodes (DLNs) were visualized at 10 min intervals.

**Results:**

A fluorescent lymph node (FLN) was detected in 19/21 dogs (90%) and was the right or left tracheobronchial lymph node (LN) in 79% of dogs. Six dogs had multiple FLNs detected simultaneously. In 4/19 dogs (21%) the first FLN(s) was the central tracheobronchial LN, sternal LN, cranial mediastinal LN or a combination of these LNs. The median time to fluorescence in the first FLN was 30 min (range: 10–50 min) for the OLV group and 10 min (range: 10–20 min) for the NV group (*p* = .0012).

**Conclusion:**

The SLN or DLN for the caudal lung in normal dogs was variable, but often included the tracheobronchial LN. One lung ventilation slowed the detection of ICG in the lymphatics.

**Clinical significance:**

Biopsy of tracheobronchial LNs for caudal lung tumors without SLN mapping may provide inaccurate staging information in some dogs. During NIRF‐guided SLN mapping, delaying OLV induction may improve SLN identification.

## 1 INTRODUCTION

Primary pulmonary neoplasia accounts for 1% of all canine tumors.^1^, ^2^, ^3^ The majority of these tumors are malignant and prognosis is strongly dependent on tumor type, grade, and stage of disease.^4^, ^5^, ^6^, ^7^, ^8^ Lymph node (LN) metastasis is an important factor in determining stage of disease and prognosis in dogs.^4^, ^5^, ^6^, ^7^, ^8^ In a recent study, median survival time of dogs with primary lung tumors without regional LN metastasis was 2.7 times longer than dogs with regional LN metastasis.^6^

Studies on sentinel LN mapping (SLNM) have shown that there is variation in lymphatic drainage amongst patients and therefore the anatomically closest LN to the tumor cannot be assumed to be the sentinel LN.^9^, ^10^ The use of near‐infrared (NIR) technology for SLNM allows for optical image‐guided surgery with real‐time visualization of lymph vessels and sentinel LNs and has gained popularity in human medicine for numerous cancers including pulmonary neoplasms.^11^, ^12^, ^13^, ^14^ The most commonly used fluorescent dye with NIR imaging is indocyanine green (ICG) due to its safety profile and affinity for binding to lipoproteins and uptake into the lymphatic system.^11^

While a previous study attempted mapping lymphatic drainage of the pulmonary parenchyma with blue dye and scintigraphy, numerous false negative LNs were missed due to limitations with dye uptake and therefore SLNM for the pulmonary parenchyma is still poorly understood in dogs.^15^ Sentinel LN mapping with ICG and NIR technology has been reported in one case report in a dog undergoing thoracoscopy for a lung tumor.^14^ The primary goal of this study was to determine the anatomic location of the sentinel LN using NIR imaging following injection of ICG into the caudal lung lobes in normal dogs. The caudal lung was selected for study because this is the most common location for primary lung tumors in dogs.^8^

During thoracoscopy in both humans and dogs, one lung ventilation (OLV) is often implemented to create working space within the pleural cavity.^13^, ^14^, ^16^, ^17^ It is unknown whether OLV and collapse of the lungs on the side of the tumor affects the diffusion of ICG from the lung into the lymphatic system in dogs. The secondary goal of this study was to determine the effect of OLV on the detection of ICG in the sentinel LN. We hypothesized that the SLN would be the same in all dogs following caudal lung lobe injection (right vs. left) with indocyanine green (ICG) and that OLV would affect the detection of ICG in the SLN in dogs.

## 2 MATERIALS AND METHODS

A total of 21 normal purpose bred Beagle dogs, belonging to a university research colony, were enrolled in the study. This study was approved by the Institutional Animal Care and Use Committee (IACUC #3650). Dogs were randomly assigned to either the one lung ventilation (OLV) group or the normal ventilation (NV) group with a coin flip. For each dog in the OLV group, a dog in the NV group was matched: right caudal versus left caudal lung lobe for injection and randomly assigned using a coin flip. Dogs were placed under general anesthesia using a standardized protocol. Dogs were premedicated with butorphanol 0.4 mg/kg IM and atropine 0.02 mg/kg IM and an IV catheter was placed in a peripheral vein. Propofol 2–6 mg/kg IV was administered to effect. Dogs were intubated, maintained on isoflurane and 100% oxygen, and mechanically ventilated using a pressure cycle ventilator for both OLV and NV groups. Ventilator settings were determined to maintain a pulse oximeter saturation (SPO_2_) above 95% and end‐tidal CO_2_ (ETCO_2_) between 40 and 45 mmHg. Positive end expiratory pressure (PEEP) and peak inspiratory pressure (PIP) were recorded. Anesthetic monitoring included direct blood pressure, SPO_2_, capnography, ETCO_2_, and electrocardiography. A mean arterial blood pressure < 60 mmHg was considered hypotension and was treated with dopamine 1–10 mcg/kg/min IV. Cefazolin was administered at 22 mg/kg IV at induction. Dogs received intercostal local anesthetic blocks with bupivacaine 0.25% at the level of T4–T8. In addition, thoracic paravertebral plane blocks were performed at the level of T5 and T9 with bupivacaine 0.25%.

The left or right hemithorax was clipped corresponding to the side of lung injection. The patient was transported to the operating room and placed in lateral recumbency with the dorsum elevated 30°. For dogs in the OLV group, a 5 or 7 french Arndt endobronchial blocker (EBB) (Cook Medical, Bloomington, Indiana) was selected based on patient size and placed in the appropriate mainstem bronchus under the guidance of a flexible endoscope (C‐MAC 8403ZX, Karl Storz, El Segundo, California). The non‐dependent lung was deflated by leaving the EBB open to the atmosphere. The patient was stabilized with the EBB in place for 15 min prior to additional procedures.

The surgical site was aseptically prepared. An intercostal thoracoscopic approach was performed using a two‐port technique. A 11.5 mm and 5.5 mm thoracoport (Medtronic, Minneapolis, Minnesota) were placed in the sixth and ninth intercostal spaces, respectively, on the side that the dog was assigned for lung injection. A 3‐D, 10 mm, 0° endoscope (TIPCAM1 Rubina, OPAL 1 NIR/ICG, Karl Storz) was introduced into the 11.5 mm cannula, and a 5 mm Babcock grasper (Karl Storz) into the 5.5 mm cannula, to allow for manipulation of the lung lobes. A 25 mg vial of lyophilized ICG was reconstituted with 10 mL of sterile water as per the manufacturer's (Diagnostic Green LLC, Farmington Hills, Michigan) instructions to 2.5 mg/mL and used within 6 h. Indocyanine green dye (2.5 mg per 1 mL) was percutaneously injected using a 20 gauge, 2.5 inch spinal needle under direct visualization with the endoscope into either the right or left caudal lung lobe (video clip S1). The caudal lung lobe was stabilized by gently grasping with the 5 mm Babcock forceps. The needle was inserted approximately 1 cm deep into the lung parenchyma. The syringe was first aspirated and then the ICG was slowly injected to minimize leakage of ICG out of the lung and into the pleural cavity. Following injection, endoscopic grasping forceps were used to apply pressure to the injection site on the pleural surface of the lung to minimize leakage. Time 0 began immediately following completion of the ICG injection. An overlay of white light and infrared light imaging was utilized (IMAGE1S Rubina, Karl Storz) to explore the hilus of the lungs and cranial mediastinum for fluorescence within a LN at 10 min intervals up to 40 min. Anatomic location of the first fluorescent lymph node (FLN) was documented along with time to detection. The SLN was defined as the first detected FLN. If multiple FLNs were detected at scheduled inspection interval, then LNs were classified as draining lymph nodes (DLNs) rather than SLN. Classification of the LNs was based on the description by Steffey et al.^18^ and Baum's Lymphatic System of the Dog.^19^ For dogs in the OLV group, the EBB was removed at 40 min and an additional lymph node explore was performed at 50 min.

A 14 french thoracostomy tube (Mila International, Florence, Kentucky) was placed. Thoracoports were removed, and a routine two layer closure was performed. The pleural space was evacuated through the thoracostomy tube and liposomal encapsulated bupivacaine (2 mL; Nocita, Elanco, Greenfield, Indiana) was administered for local analgesia at the cannula sites. The thoracostomy tubes were removed prior to extubation if negative pressure was achieved. All dogs were given carprofen 2.2 mg/kg subcutaneously.

No dogs were euthanized as a result of this study. Dogs recovered from anesthesia and were monitored overnight. Postoperative pain management included carprofen 2.2 mg/kg orally every 12 h for 3 days and gabapentin 10 mg/kg orally every 12 h for 3 days. Dogs were returned to socialized housing the following day, and the incisions were checked twice daily for 3 days. Dogs were rechecked for suture removal 10 days postoperatively.

### 2.1 Statistical analysis

Data were assessed for normality using a goodness of fit test. Normally distributed continuous data are reported as mean and SD and data not normally distributed are reported as median and range. Nonparametric tests were used for comparison tests for data not normally distributed, including Wilcoxon rank‐sum test, Chi‐square analysis, and Fisher's exact test. From time 0 to 50 min MAP, ETCO_2_, SPO_2_, PEEP, PIP, and dopamine dose were recorded and the median for each dog during this time frame was documented to allow for comparison of variables between OLV and NV groups. Kaplan–Meier analysis was used to analyze time to first lymph node fluorescence, censoring those that did not fluoresce or fluoresced after the endobronchial blocker was removed. Log‐rank test was used to compare distributions between groups. Statistical significance was set at *p* < .05. Statistical analyses were conducted using JMP (JMP18.2.2, SAS, Cary, North Carolina).

## 3 RESULTS

A total of 21 purpose bred Beagles were included in this study. There were 18 intact females and three castrated males. Median age was 1 year (range: 1–5). Mean weight was 8.19 kg ± 2.07 kg. There were 12 dogs in the OLV group and nine dogs in the NV group. There was no difference in age or weight between groups (Table 1). A total of 11 dogs were injected with ICG in the left caudal lung lobe and 10 dogs were injected in the right caudal lung lobe (Figure 1). Overall median anesthesia time was 150 min (range: 113–235) and overall surgery time was 75 min (range: 56–153). There was no difference in anesthesia or surgery time between groups, *p* = .337 and *p* = .051, respectively (Table 1). Dopamine was used for blood pressure support in 18/21 (86%) dogs. There was no difference in the median dopamine dose between dogs with OLV and NV (*p* = .771). Respiratory parameters were compared between OLV and NV groups and there was no difference in median MAP, SPO_2_, or ETCO_2_. There was no difference in the median PEEP or PIP used between dogs with NV and OLV (Table 2).

**TABLE 1:** Perioperative parameters for dogs with OLV and NV. Continuous variables are expressed as median and range or mean ± SD.

Parameter	OLV (12)	NV (9)	Overall (21)	*p*‐value
Weight (kg)	8.45 ± 1.60	7.83 ± 2.64	8.19 ± 2.07	.51
Age (years)	1 year (1–4)	1 year (1–5)	1 year (1–5)	.962
Anesthesia time (min)	180 (113–235)	147 (119–185)	150 (113–235)	.337
Surgery time (min)	86 (65–153)	64 (56–120)	75 (56–153)	.051

Abbreviations: NV, normal ventilation; OLV, one lung ventilation;

**FIGURE 1:**
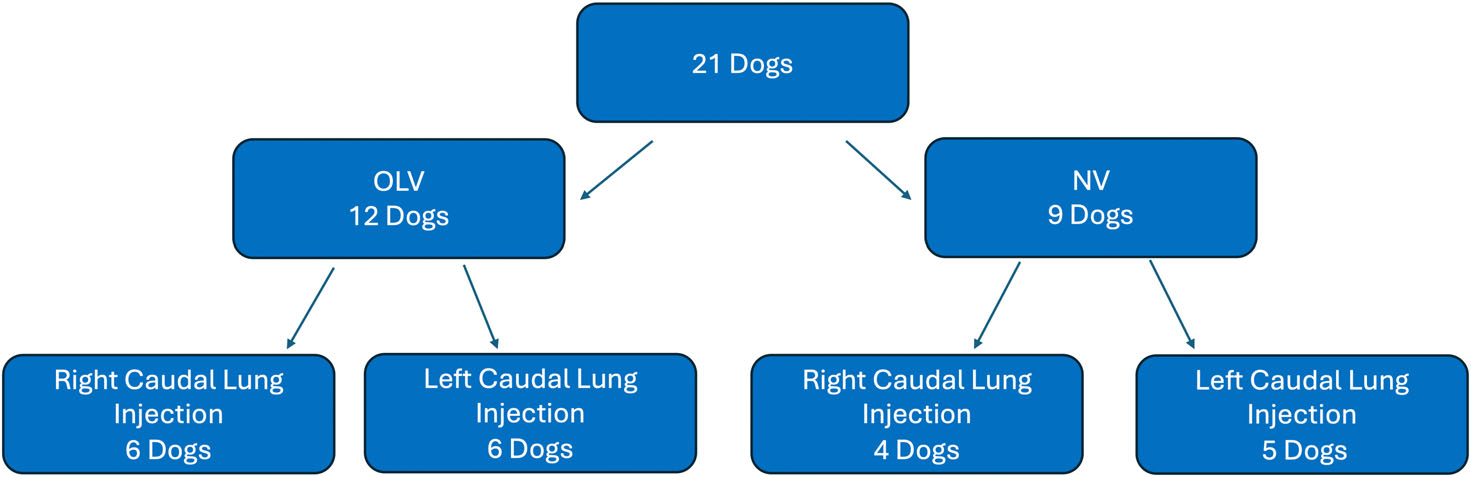
Study population. NV, normal ventilation; OLV, one lung ventilation.

**TABLE 2:** Respiratory parameters for dogs with OLV and NV from ICG injection to 50 min post‐ICG injection. Median (range).

Parameter	OLV (12)	NV (9)	*p*‐value
Mean arterial pressure mmHg	75 (65–89)	80 (67–108)	.226
Median dopamine dose (mcg/kg/min)	2.75 (0–10)	6 (0–7.5)	.771
ETCO_2_ (mm Hg)	45 (40–74)	54 (34–58)	.374
SPO_2_ (%)	97.5 (90–100)	96 (95–99)	.352
PEEP	2 (0–3)	0 (0–3)	.521
PIP	10 (0–15)	7 (0–11.5)	.291

Abbreviations: ETCO_2_, end‐tidal carbon dioxide; ICG, indocyanine green; NV, normal ventilation; OLV, one lung ventilation; PIP, peak inspiratory pressure; PEEP, positive end expiratory pressure; SPO_2_, pulse oximeter saturation.

A FLN was detected in 19/21 (90.5%) of dogs (Figure 2; Table 3). Of the 19 dogs where a FLN was detected, a SLN was identified in 13 (68%) dogs. The left or right tracheobronchial LN was the SLN in 10 (77%) of 13 dogs. The SLN was the central tracheobronchial LN, cranial mediastinal LN, and sternal LN in one dog each (Table 3). Multiple fluorescent LNs were detected in six (32%) of 19 dogs and were classified as DLNs. The tracheobronchial and cranial mediastinal LNs were the DLNs in five dogs and the cranial mediastinal and sternal LN were the DLNs in one dog. A total of 13 dogs (68%) had a single FLN while three dogs had two FLNs, two dogs had four FLNs and one dog had three FLNs. There was no difference in the location of FLNs following right versus left lung injection (*p* = .45, *p* = .944, respectively).

**FIGURE 2:**
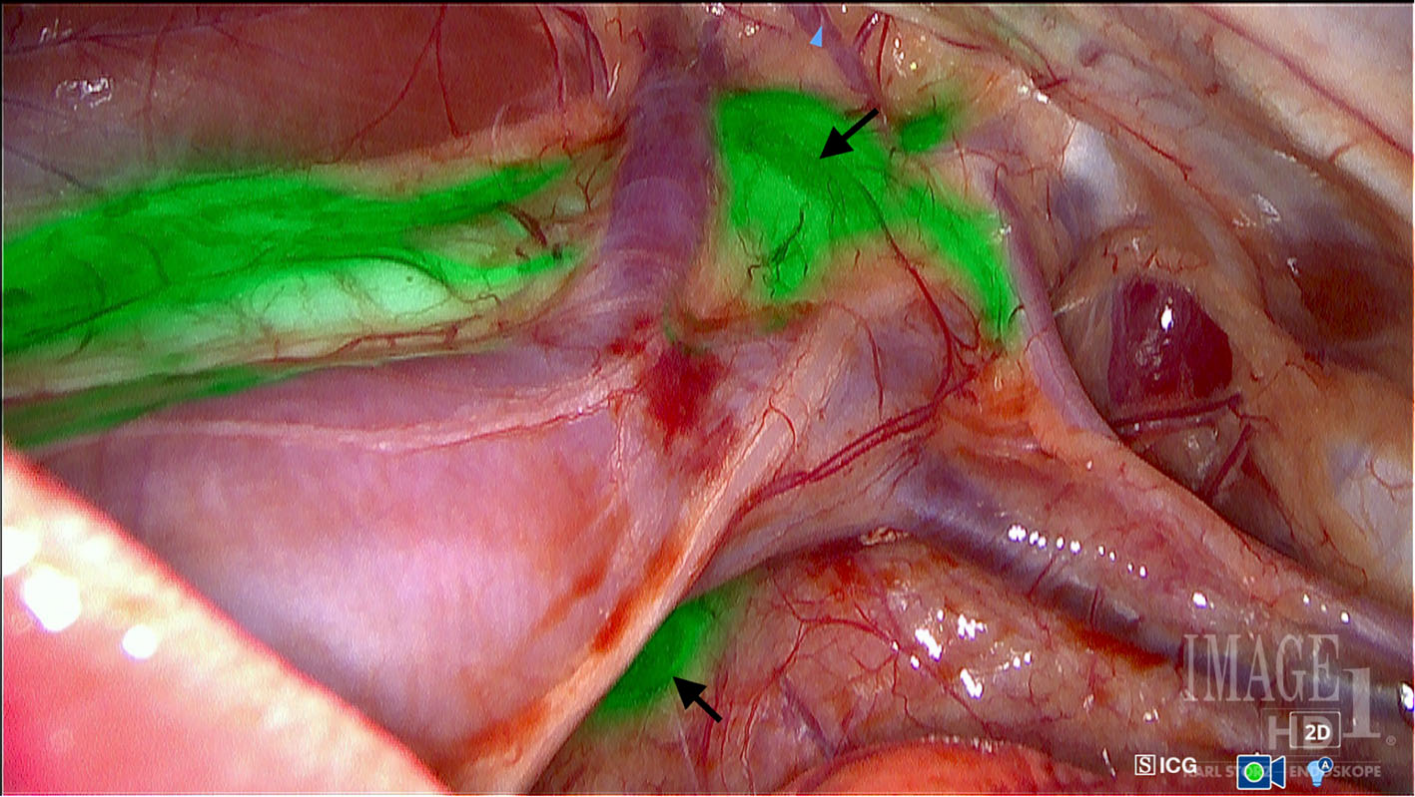
Fluorescence seen within lymphatic tracts and two cranial mediastinal lymph nodes (black arrows) after right caudal lung injection with indocyanine green.

**TABLE 3:** Distribution of first fluorescent lymph node in dogs with right lung injection versus left lung injection.

Draining lymph node	Right lung injection 10 dogs	Left lung injection 11 dogs
None	1	1
Right or left TB	3	7
Central TB	1	0
Cranial mediastinal	1	0
Sternal	0	1
Right or left TB and cranial mediastinal	3	2
Cranial mediastinal and sternal	1	0

Abbreviation: TB, tracheobronchial.

All nine dogs in the NV group had a FLN detected while only eight of 12 dogs in the OLV group had a FLN detected with the EBB in place (*p* = .104). In two of the dogs where a FLN was not detected at 40 min in the OLV group, fluorescence was seen at 50 min after the EBB was removed. Median time to FLN detection was 30 min (95% CI: 10 min to not estimable) in the OLV group and 10 min (95% CI: 10–20 min) in the NV group (*p* = .0012, Figure 3). Median time to LN fluorescence after a left caudal lung injection was 10 min (95% CI: 10–30 min)and after a right caudal lung injection was 20 min (95% CI: 10–30 min) (*p* = .587, Figure 4).

**FIGURE 3:**
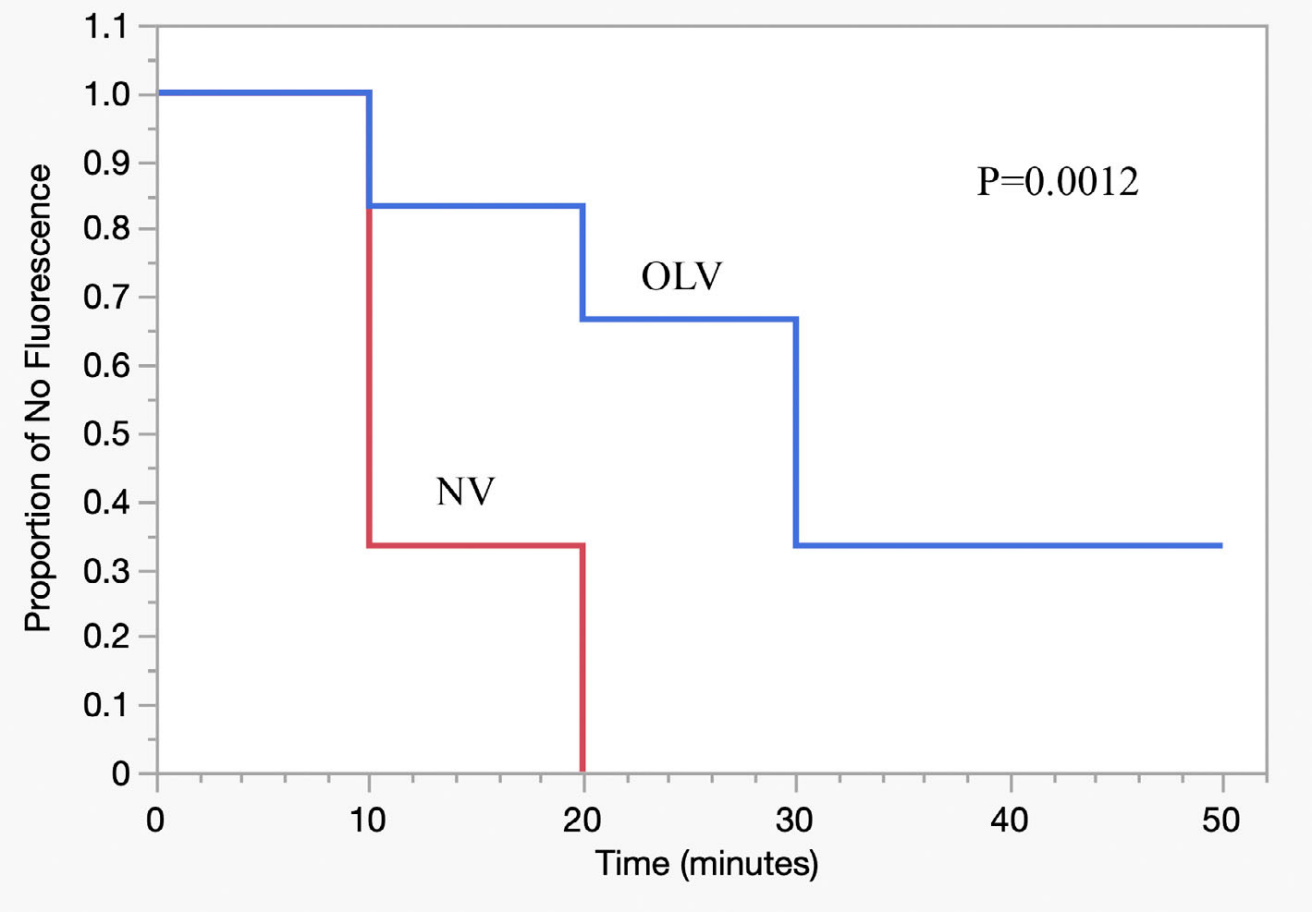
The effect of one lung ventilation (OLV) on time to fluorescence of the first lymph node(s).

**FIGURE 4:**
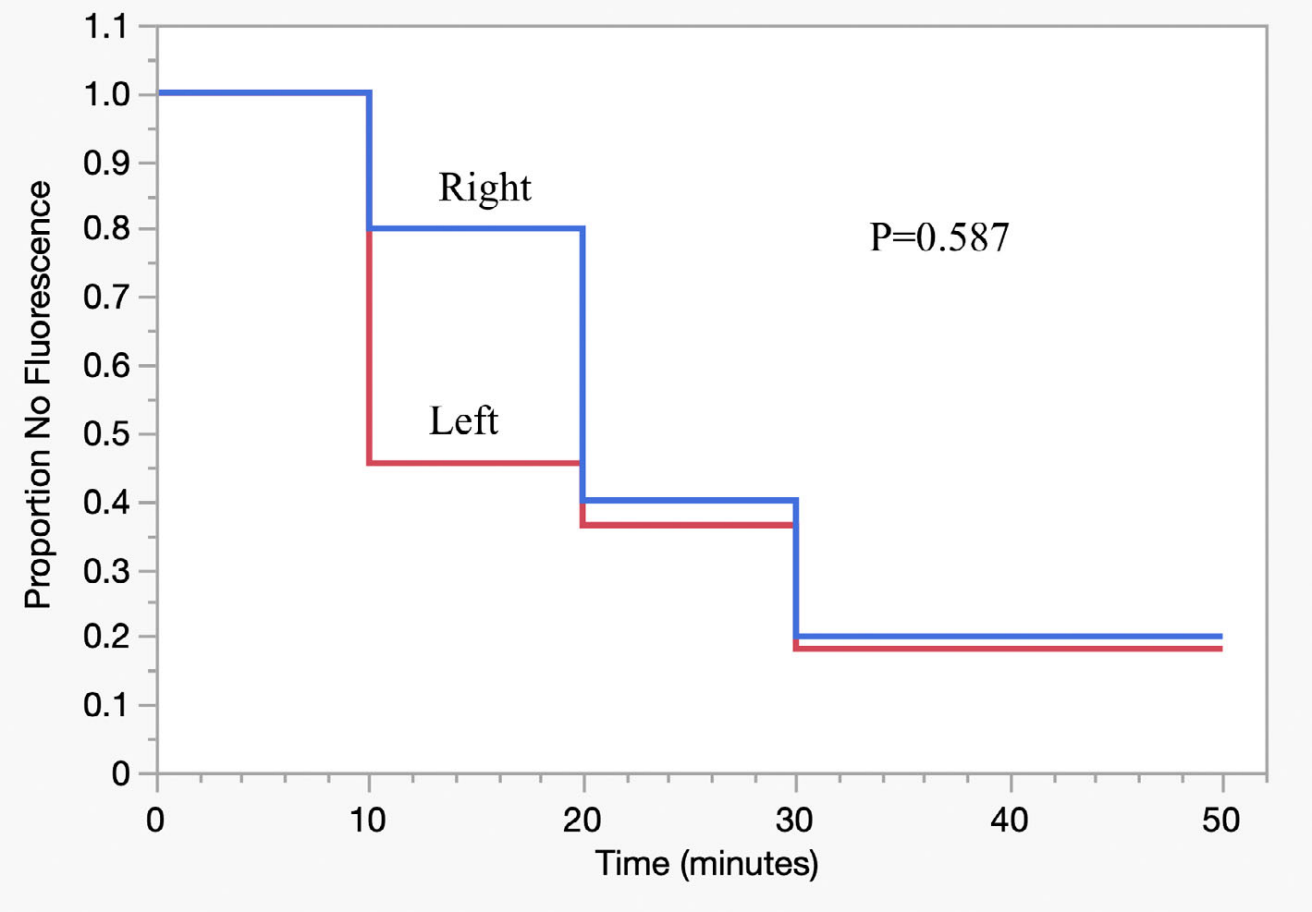
The effect of laterality of injection on time to fluorescence of the first lymph node(s).

## 4 DISCUSSION

Fluorescent lymph nodes were identified in most dogs following caudal lung lobe injection. A SLN was identified in 68% of dogs and was most commonly the left or right tracheobronchial LN (77%). In dogs where DLNs were identified, but a single SLN could not be determined, the tracheobronchial LN was a DLN in 83% of those dogs. The tracheobronchial LN was neither a SLN nor a DLN in 21% of dogs. Furthermore, in dogs where only DLNs were detected, it is possible that the tracheobronchial LN was not the true SLN given that imaging of the LNs was performed at 10 min intervals and the first LN to fluoresce may have been missed. While the location of the first fluorescent LN(s) following left or right caudal lung injection with ICG was variable; injection side did not influence FLN location.

Lymphatic drainage in the thorax is poorly understood. Attempts have been made at adopting the human classification scheme.^15^ We attempted to further classify the location of the SLNs that were found in the cranial mediastinum, but found that there was significant variation from dog to dog which prevented us from providing a more detailed descriptive location other than the cranial mediastinal region. Additional studies are necessary to better describe the lymphatic drainage within the thorax.

The second objective of the study was to determine the effect of OLV on the detection of ICG in the first FLN(s). Fluorescence was visualized in LNs in all dogs with NV, while a FLN was visualized in only eight of 12 dogs in the OLV group while the EBB was in place. When ICG is injected with other SLNM applications local massage of the region is recommended.^20^, ^21^, ^22^ This pressure is thought to facilitate lymphatic uptake and passage to the draining LNs. With OLV the lungs are blocked from ventilation, preventing inflation and deflation. Ventilation of the lungs may provide a similar mechanical effect to peritumoral massage in other anatomic locations; further evaluation is needed. Four dogs in the OLV group did not have a FLN with the blocker in place and only two of those dogs had a SLN visualized once the blocker was removed. This finding has clinical applications as OLV during thoracoscopy is often induced prior to surgery once the patient is positioned in the operating room. If OLV is induced prior to lung injection with ICG during SLNM, then detection of the SLN may be delayed or inhibited all together.

This study had numerous limitations. The ideal dosage of ICG for this application is unknown. The dose of 2.5 mg was chosen by the authors due to previous experience with SLNM in clinical cases with lung tumors. Previous clinical experience revealed difficulty of SLNM with ICG during thoracoscopy and the authors wanted to ensure that failure of the SLNM was not due to a low dose of ICG. The caudal lung lobe was selected in this study since it is the most common location of primary pulmonary neoplasia in dogs.^8^ With only 21 dogs in the study, it was not feasible to inject additional lung lobes for SLNM. It is possible that lymphatic drainage from other lung lobes varies. Leakage of ICG occurred in some dogs following lung injection. It is possible that this influenced the SLN location or failure to identify a single SLN. Normal purpose‐bred beagles were used in this study. Lymphatic drainage may differ with breed and when pulmonary neoplasia is present. A single injection of ICG was used in this study similar to Griffin et al.^14^ Results may differ with a four quadrant injection. An intercostal thoracoscopy approach was used in this study. This approach is commonly performed in a clinical setting for thoracoscopic or open lung lobectomy and therefore was used in the study. However, it is possible that lymphatic drainage crosses to the contralateral hemithorax and the SLN may have been missed in some cases since ICG imaging was limited to the ipsilateral hemithorax. While positioning in dorsal recumbency for subxiphoid transdiaphragmatic thoracoscopy would have permitted bilateral access, it would have limited visibility of the hilar region for LN assessment. The thorax was explored at 10 min intervals for FLNs. At some intervals, multiple FLNs were noted simultaneously and therefore were classified as DLNs rather than SLNs. Draining LNs contain the SLN, but in this study we were unable to determine the exact SLN. While multiple SLNs can exist, without real‐time assessment we were only able to classify these LN as DLNs. Due to the manipulation of lung lobes required to visualize the different LNs, it was not possible to continuously visualize all thoracic LNs. A standardized anesthetic protocol with local blocks were used to minimize variability in physiologic variables during SLNM. There were no differences between groups for the anesthetic parameters or physiologic variables that were measured; however, given the small number of dogs in the study, it is possible that systemic variables may have influenced time to fluorescence.

The SLN for the caudal lung in normal dogs is variable, but often includes the tracheobronchial LN. Routine biopsy of the right or left tracheobronchial LNs in dogs with primary lung neoplasms in the caudal lung lobe may result in inaccurate staging information in some dogs. Real time SLNM with ICG and NIR technology may provide more accurate information on the location of the SLN. If SLNM with ICG is performed with thoracoscopy, it may be prudent to delay induction of OLV until after peritumoral ICG injection to increase the likelihood of identifying the SLN. Further evaluation of the delayed induction of OLV is necessary to determine whether this approach improves SLN detection.

## AUTHOR CONTRIBUTIONS

Marvel SJ, DVM, MS, DACVS (Small Animal): Contributed to design of the study, assisted in surgical management of cases, analyzed data for statistical significance, drafted and revised manuscript. Kaschark N, DVM: Contributed to design of the study, assisted in surgical management of the cases, interpreted data, and revised the manuscript. Boscan P, DVM, DACVAA: Contributed to design of the study, assisted in anesthetic management of the cases, and revised the manuscript. Monnet E, DVM, DACVS, DECVS: Contributed to design of the study, was responsible for the surgical management of cases, analyzed data for statistical significance, drafted and revised manuscript. All authors provided critical review of the manuscript and endorse the final version. All authors are aware of their respective contributions and have confidence in the integrity of all contributions.

## CONFLICT OF INTEREST STATEMENT

The authors declare no conflict of interest.

## Supporting information


**Video S1:** Lung injection with ICG.

## Data Availability

The data that support the findings of this study are available on request from the corresponding author. The data are not publicly available due to privacy or ethical restrictions.
